# Impact of Long-Term Depression on Employment Outcomes: A Systematic Review and Case Series From Iraq on Career Trajectory and Job Stability

**DOI:** 10.7759/cureus.70755

**Published:** 2024-10-03

**Authors:** Hasan I Alshammaa, Rania H Al-Taie, Abdallah M Mujbel

**Affiliations:** 1 Department of Psychiatry, Al-Yarmouk Teaching Hospital, Baghdad, IRQ; 2 Department of Surgery, College of Medicine, Mustansiriyah University, Baghdad, IRQ; 3 Department of Psychiatry, College of Medicine, Mustansiriyah University, Baghdad, IRQ

**Keywords:** career trajectory, depression, employment outcomes, iraq, socioeconomic impact

## Abstract

Background

Long-term forms of depression, especially chronic and episodic, make it very hard for any individual to maintain a steady job or develop in his/her workplace, which reduces the ability to gain financial security. The purpose of this study is to investigate and thoroughly examine the impact of long-term depression on career trajectories and job stability using a methodical evaluation of the literature supplemented with case studies.

Methodology

This study combined a systematic review of available literature with a detailed case series analysis. The literature search was conducted systematically in three major databases, namely, PubMed, PsycINFO, and Scopus. The systematic review synthesized findings from studies that assessed the relationship between chronic and episodic long-term depression and employment-related outcome measures, i.e., job stability, upward career mobility, and socioeconomic status. The studies published between 2000 and 2024 were included and qualified. The case series contributed qualitative depth using eight personal experiences illustrating how the use of self-workplace dynamics interacted with depressive symptoms to shape employment.

Results

The systematic review provided consistent evidence that depression negatively influences employment status, such as decreased income and an increased rate of unemployment and disability claims. The current investigation included 29 studies, which were chosen after a rigorous screening process that included identifying 10,651 records and removing irrelevant or duplicate entries. The case series underlined further that it is the role of support executed by the workplace and societal stigma that mitigates or exaggerates these outcomes. In cases, people whose careers were disrupted by depression (job loss, low productivity, and long-term financial pressure) evidenced a huge change.

Conclusions

The effects of chronic and episodic long-term depression interfered with employment and socioeconomic well-being and, in fact, expanded beyond the individual to affect larger societal factors. Healthcare providers should collaborate with employers to ensure affected individuals receive appropriate accommodations in the workplace along with responses to mental health concerns. Further, policymakers should create inclusive policy environments to address the demands of people concerning job security and access to mental health related to depression. In addition, they should promote anti-stigma campaigns targeted at the reduction of societal and workplace discrimination against mental health issues.

## Introduction

Depression is known to be one of the most critical mental health issues whereby a person experiences extended periods of low mood and significant impairment in everyday life, especially chronic depression and episodic long-term depression [1]. Chronic depression denotes conditions such as persistent depressive disorder characterized by continuous symptoms that persist for years, leading to socioeconomic consequences that may be serious and persistent. Episodic long-term depression, while interspersed with periods of remission, also disrupts daily life and career advancement, especially when depressive episodes recur frequently over time [1].

A complex pathophysiology in the biological, psychological, and social domains underlies both chronic and episodic long-term depression. Molecular investigations have identified a role for neurotransmitter imbalance, dysregulation in the hypothalamic-pituitary-adrenal axis, and genetic predispositions in the persistence and recurrence of depressive symptoms [2]. Moreover, these types of depression have a deep economic and social cost, especially in terms of effects on employment/career outcomes.

The challenges of stable employment, building careers, or establishing an independent financial life are often hard to meet for those experiencing chronic or episodic long-term depression. It is enhanced further by the continuous or recurrent character of depressive states, which reduces work productivity and raises absenteeism and disability claims that negatively affect career trajectory and job stability [1,2]. The risk is the greatest for those with more severe or recurrent episodes. On the other hand, women compared to men are affected more by the link between unemployment and depression. These facts further underline the critical intersection between depression and employment [3].

In fact, recent research has demarcated that the long-term psychological impact of global crises such as the COVID-19 pandemic extended beyond the recovery period. For example, a study conducted among survivors 40 months after the infection in the Kurdistan region of Iraq showed moderate anxiety and mild depression with normal stress. The results indicate that targeted mental health interventions and support need to be urgently implemented in both short- and long-term contexts [4].

Cognitive-behavioral therapy plays a very important role in the treatment of persistent depressive disorder and dysthymia. It is highly effective in dealing with the chronic nature of these mood disorders. cognitive-behavioral therapy helps individuals identify negative thought patterns and behaviors that contribute to their depression. Because it is a tailored therapy, and modular, treatment options may be less clear-cut, cognitive-behavioral therapy appeals to the multifaceted symptoms of persistent depressive disorder. It has been shown to improve both short-term and long-term outcomes in patients [1].

Keeping in mind the larger individual and social perspective of chronic and episodic long-term depression, there is a need to understand their effects on employment outcomes beyond unemployment. Therefore, this paper aims to fill this gap through an integration of a structured literature review with detailed case studies.

## Materials and methods

This is a mixed-methods study that involves the systematic review of available litterateurs, as well as an in-depth case series. The systematic review was conducted to summarize available evidence on how chronic and episodic long-term depression affects employment outcomes pertaining to career trajectory, job stability, and long-term socioeconomic consequences. This is complemented by the case series in providing an in-depth qualitative analysis of single cases to understand the real-world implication of such findings within diverse personal and professional contexts.

Case series

Case Selection

Eight cases were selected in light of their relevance to the study’s objective and demographic, clinical history, employment record, and socioeconomic diversity. Drawing from clinical records and patient interviews ensured that cases covered a full range of different experiences with long-term depression.

Data Collection

For every case, detailed information was gathered on patient demographics, clinical history, employment history, impact on socioeconomic status, psychosocial and cultural factors, coping strategies, workplace adaptations, and long-term outcomes. Data for each case were collected based on patient reports and clinical records.

Analysis of Case Data

Thematic analysis of the case data identified major themes and patterns across cases to highlight common challenges and coping mechanisms related to depression and employment. This then was mapped together with the results from the systematic review, pointing out places of convergence and divergence between individual experiences documented in the case series and general trends of the studies.

Ethical Considerations

The investigation was conducted according to the principles outlined in the Helsinki Declaration. Informed consent was obtained from all case series patients, and their anonymity was secured by anonymizing all of their data to be analyzed. Concerning the protection of research subjects, the research reduced potential risks for participants and ensured respect for the rights and well-being of all participants during the conduct of the research.

Integration of Findings

The systematic review and case series findings are integrated qualitatively in the discussion section to develop a coherent understanding of the effects of long-term depression on employment outcomes. This methodology brings out broad trends while giving detailed insights into the individual experience and gives a more holistic view of the different challenges that people suffering from chronic and episodic long-term depression face in the workplace.

Systematic review

Search Strategy and Information Sources

**Figure 1:**
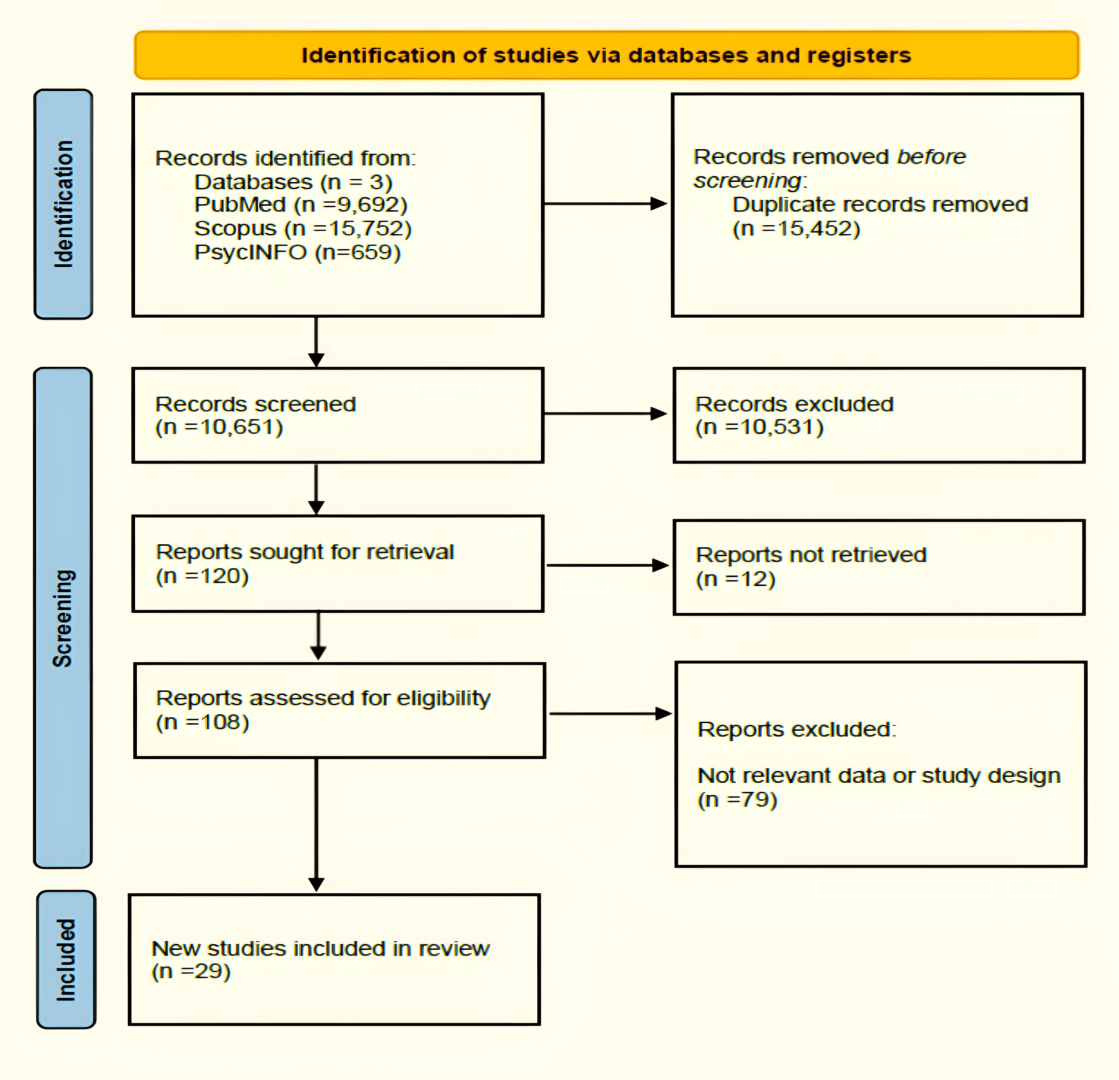
Preferred Reporting Items for Systematic Reviews and Meta-Analyses (PRISMA) flowchart of the included studies.

This systematic review was carried out in accordance with the Preferred Reporting Items for Systematic Reviews and Meta-Analyses (PRISMA) guidelines (Figure 1) [5]. A comprehensive search strategy was developed to retrieve relevant literature. Key search terms included “depression,” “major depressive disorder,” “career,” “job,” and “employment.” These terms were combined using Boolean operators to maximize the retrieval of pertinent studies. The search process was meticulously documented to ensure transparency and replicability. The literature search was systematically conducted in three key databases, namely, PubMed, PsycINFO, and Scopus. Terms played an important role in designing this search strategy with the aid of Boolean operators to identify studies focused on one or more aspects related to the impact of depression on career outcomes.

Eligibility Criteria

In the review, studies involving participants diagnosed with chronic depression or episodic long-term depression were covered. The studies had to consider career-related outcomes that included attaining jobs, job performance, rising through careers, income, and satisfaction attained in jobs. The studies considered had to entail quantitative, qualitative, and mixed-method designs. only peer-reviewed articles, theses, and dissertations from 2000 through 2024 were eligible.

Studies that focused on other psychiatric conditions without putting specific emphasis on depression were excluded. Further, non-peer-reviewed articles, conference abstracts, and general opinion pieces were also excluded. Moreover, studies of patients with some other physical morbidities, such as multiple sclerosis or epilepsy, and those examining the impact of employment on depression rather than the effect of depression on career outcomes, were not taken into consideration.

Screening Process

Screening was done in the following two stages: title and abstract screening, followed by full-text screening. In the first stage, the titles and abstracts of all retrieved articles were screened by two independent reviewers for relevance according to pre-established inclusion and exclusion criteria. Any discrepancies between these two reviewers were resolved through discussion or, in case of necessity, with a third reviewer. To manage the screening process efficiently, the Rayyan website was utilized, allowing reviewers to collaborate effectively and resolve conflicts during the screening and selection process.

Data Extraction

Data extraction was performed using a standardized data extraction form designed to capture essential information from each study. The extracted data included the author(s) and year of publication, study design, population and sample size, measures of depression, career outcomes assessed, key findings, and any noted study limitations. Two independent reviewers conducted the data extraction process, ensuring accuracy and consistency. Any discrepancies in data extraction were resolved through discussion. In the second stage, the full texts of the studies that passed the initial screening were reviewed in detail to assess their eligibility.

Quality Assessment

The quality of the included studies was subsequently appraised using the Risk of Bias in Non-randomized Studies-Interventions (ROBINS-I) (Table 1) [6]. The tool assesses several domains, such as bias due to confounding, bias in the selection of participants, classification of interventions, deviations from intended interventions, missing data, measurement of outcomes, and selection of the reported result.

**Table 1:** ROBINS-I assessment of studies on depression and employment outcomes.

Study ID	Authors	Confounding	Selection of patients	Classification of interventions	Deviations from intended interventions	Missing data	Measurement of outcomes	Selection of reported results
1	Holma et al. (2012) [7]	Low to Moderate	Low	Low	Low	Moderate	Low	Low to moderate
2	Hakulinen et al. (2019) [8]	Moderate	Low	Low	Low	Moderate	Low	Low to moderate
3	Chen et al. (2023) [9]	Moderate	Low	Low	Low	Moderate	Low	Low
4	Petersen et al. (2022) [10]	Moderate	Low	Low	Low	Moderate	Low	Low to moderate
5	Rizvi et al. (2015) [11]	Moderate	Low	Low	Low	Moderate	Low	Low
6	Ringdal and Rootjes (2022) [12]	Moderate	Low	Low	Low	Moderate	Low	Moderate
7	Elinson et al. (2004) [13]	Moderate	Low	Low	Low	Moderate	Low	Moderate
8	Gilmour et al. (2007) [14]	Moderate	Low	Low	Low	Moderate	Low	Low
9	Andreeva et al. (2015) [15]	Moderate	Low	Low	Low	Moderate	Low	Low
10	Druss et al. (2001) [16]	Moderate	Low	Low	Low	Moderate	Low	Moderate
11	Witt et al. (2021) [17]	Moderate	Low	Low	Low	Moderate	Low	Low
12	Campbell et al. (2022) [18]	Moderate	Low	Low	Low	Moderate	Low	Low
13	Heinz et al. (2018) [19]	Moderate	Low	Low	Low	Low	Moderate	
14	Thielen et al. (2013) [20]	Moderate	Low	Low	Low	Moderate	Low	Low
15	Prieto-Vila et al. (2024) [21]	Moderate	Low	Low	Low	Moderate	Low	Moderate
16	Patten et al. (2009) [22]	Moderate	Low	Low	Low	Moderate	Low	Moderate
17	Rottinghaus et al. (2009) [23]	Moderate	Low	Low	Low	Moderate	Low	Moderate
18	Zivin et al. (2012) [24]	Moderate	Low	Low	Low	Moderate	Low	Low
19	Naicker et al. (2013) [25]	Moderate	Low	Low	Low	Moderate	Low	Low
20	Lee et al. (2016) [26]	Moderate	Low	Low	Low	Moderate	Low	Moderate
21	Bubonya et al. (2019) [27]	Moderate	Low	Low	Low	Moderate	Low	Moderate
22	Woodhead et al. (2020) [28]	Moderate	Low	Low	Low	Moderate	Low	Moderate
23	Dobson et al. (2024) [29]	Moderate	Low	Low	Low	Moderate	Low	Low
24	Lee et al. (2020) [30]	Moderate	Low	Low	Low	Moderate	Low	Moderate
25	López-López et al. (2019) [31]	Moderate	Low	Low	Low	Moderate	Low	Low
26	Lamberg et al. (2010) [32]	Moderate	Low	Low	Low	Moderate	Low	Low
27	Lerner et al. (2004) [33]	Moderate	Low	Low	Low	Moderate	Low	Moderate
28	Huijs et al. (2017) [34]	Moderate	Low	Low	Low	Moderate	Low	Moderate
29	Veronese et al. (2012) [35]	Moderate	Low	Low	Low	Moderate	Low	Low

Data Synthesis

Synthesis used a qualitative thematic analysis approach in the studies identified with qualitative data. According to age, sex, type of employment, and degree of depression, subgroup analyses were done to identify possible modifying effects of the influence of depression on career outcome. These findings were synthesized to provide an overview of how chronic and episodic long-term depression relate to different employment-related outcomes.

## Results

Case series presentation

This case series on eight individuals suffering from chronic and episodic long-term depression helps to put into perspective how this condition impacted their employment, socioeconomic status, and quality of life in the real world. Each case reports a particular story about issues experienced and strategies adopted to cope with mental health problems (Table 2).

**Table 2:** Summary of case series on long-term depression and employment outcomes.

Case	Age	Gender	Type of depression	Severity (major depression inventory)	Duration of episodes	Occupation	Impact on job performance	Supportive work environment	Income change	Coping strategies	Current employment status	Long-term career impact
1	26	Male	Episodic long-term	Severe	3–6 months	Resident pharmacist (governmental)	Decreased productivity, increased absenteeism	No	Decreased to minimum	Exercise, seeking expert support	Part-time employment	Loss of full-time job
2	29	Male	Chronic	Severe	3–6 months	Practitioner pharmacist	Decreased productivity, increased absenteeism	No	Decreased to minimum	Seeking support, cognitive changes	Full-time employment (two jobs)	Slowed career development
3	27	Female	Episodic long-term	Severe	1–3 months	Government pharmacist	Decreased productivity, left second job	Yes	Decreased by 60%	Crying, sleep	Part-time employment	Loss of full-time job
4	25	Female	Episodic long-term	Severe	3–6 months	Dentist (governmental and private)	Decreased productivity, increased absenteeism	No	Decreased to minimum	Exercise, seeking support	Part-time employment	Loss of full-time job
5	34	Female	Episodic long-term	Severe	3–6 months	CEO, documentary production	Delayed studies, loss of project opportunities	No	Decreased by 70%	Exercise, seeking expert advice	No change	Loss of projects, delayed development
6	31	Female	Chronic	Moderate to severe	Continuous until treatment 3 months ago	Unemployed (formerly employed)	Lost job, unable to return to work	No	Decreased to nothing	Exercise, delayed seeking support	Unemployed	Loss of full-time job, difficulty starting new job
7	37	Female	Episodic long-term	Severe	2–5 months	Practitioner Dentist	Stopped private practice, maintained government employment	Yes	Decreased to minimum	Exercise, support from friends	Full-time employment	Mood instability affecting private practice
8	27	Female	Episodic long-term	Severe	3–6 months	Pharmacist	Decreased productivity, increased absenteeism, sick leaves	Mixed (some support, some not)	Decreased to minimum	Seeking support	Part-time employment	Loss of full-time job

Case 1: Male, 26, Resident Pharmacist

This 26-year-old male pharmacist with a diagnosis of severe episodic long-term depression, diagnosed in 2021, was facing significant problems in his personal and professional life. The diagnosis was made when he was a student, but his transition into the workforce as a resident pharmacist was marred by his deteriorating mental health. Depressive episodes lasted three to six months, causing a large decrease in productivity and an increase in absenteeism, making his duties very stressful. Although he was trying to hold on to several jobs, the unsupportive work environment added to his problems and caused him to lose his second job. His financial situation deteriorated very fast, his income was reduced to a minimum, and he was forced to live on his savings, which soon ran out. Coupled with the stigma associated with his condition and societal pressures, he did not seek help early enough and found himself lonely with little or no social support. He had only two coping mechanisms available to him: doing some exercises and talking with a professional, which both provided some amelioration but not the return of his quality of life. He is at present working part-time and is plagued by feelings of inferiority and a feeling of failure. He is still under treatment that includes medication and psychotherapy, whose principal emphasis is now on the management of his depression. Another comorbidity that the patient is suffering from is celiac disease.

Case 2: Male, 29, Practitioner Pharmacist

This 29-year-old male pharmacist has been experiencing chronic depression since 2021. Before his diagnosis, he could easily work at a primary job and even hold a second job. His mental health began interfering with his performance shortly after. As his depression was chronic, his productivity started falling, and he began to be absent more often from work. His colleagues did not know about his condition and, therefore, he got no support, and unreasonable expectations were, in fact, placed on him at work. The depression led to his having to give up the second job. This had a severe effect on his income and caused financial stress. Although the situation was not favorable in his case, he still managed to find an environment with diminished responsibilities, which allowed his workload to be better controlled. His personal life contributed very significantly to improving his health, especially the help of his wife. Gradually, through medication and psychotherapy, he improved and could resume full-time work, though it slowed down his growth in the career field.

Case 3: Female, 27, Pharmacist

This 27-year-old female pharmacist has been suffering from episodic long-term depression since 2020. The case was treated with both medicine and psychotherapy. Before diagnosis, she was working two jobs, one full-time in the pharmacy and a second shift at the same pharmacy. With a depression burden for one to three months episodically, it became extremely problematic to continue working in such a manner as her productivity had fallen completely; she finally quit her second job. Fortunately, she was working in a very supportive environment and her colleagues did understand what was happening to her, so she could keep her primary job part-time.

However, her coping mechanisms were not enough, as she resorted mostly to crying and sleeping, further impairing her functioning at work and in her personal life. The financial burden was immense because the income was reduced by 60%, and thus savings became depleted while non-essential things could no longer be afforded. Even though she received strong social support from her friends and husband, her attitude remained quite pessimistic toward recovery. She continued to work part-time and she is on treatment, but the feelings of hopelessness and fear of never achieving success do not leave her.

Case 4: Female, 25, Dentist

In 2021, a 25-year-old female dentist was diagnosed with severe episodic long-term depression and developed severe disturbances in her career. The patient is a known case of celiac disease. Before her case was diagnosed, the patient was a student. However, as she took up professional practice, her life was significantly affected because of the recurrent depressive episodes that usually stretched from three to six months. This became a hindrance to her managing her duties, which resulted in her being unable to keep a job, and she was financially at the lowest level of income. She was in an unsupportive working environment which only added more problems to her case, and her workmates did not even recognize her state of affairs. She has been in treatment but still endures very low self-esteem and failures, which has greatly affected her well-being. She is currently working part-time in a very basic capacity, looking to just scrape by and remain in treatment, which includes medication and psychotherapy with the goal of managing her symptoms.

Case 5: Female, 34, CEO of a Documentary Production Company

This 34-year-old CEO had suffered episodic long-term depression since 2006, which most deeply influenced her working life. High-severity depressive episodes ran in cycles of three to six months, delaying three years of school development and professional development, and loss of many projects. Although she held a very senior position, the stigma associated with her mental health and lack of support from her workplace increased her problems, which caused extreme economic hardship. She lost 70% of her income and all her savings. The support was meager; only her husband and one close friend showed understanding of her condition. Her depression reached a point where she considered suicide and abandoned her ambitions to build her career. Although she stays in therapy and gets to work each day at her documentary production company, things are still precarious for her career. She hopes to regain people’s trust and sign on new projects.

Case 6: Female, 31, Former Employee in the Tech Industry

This 31-year-old female was diagnosed with chronic depression in 2018 and completely lost her career. The patient also suffered from fibromyalgia. Initially working in a tech company, moderate-to-severe depressive symptoms led to job loss, and new employment could not be held onto. She became incapable of working due to major depression, further exacerbated by fibromyalgia, which made her dependent on her family financially. Although treatment was initiated just three months ago, she is experiencing a very slow recovery and much difficulty in trying to return to work. She felt isolated by the lack of support and understanding from her family, which contributed to her isolation, and has been unable to resume her normal quality of life. Her attention remains on recovery and she hopes eventually to be able to return to work.

Case 7: Female, 37, Dentist

This 37-year-old female dentist has suffered from very severe episodic long-term depression since 2016. She maintained her job with the government but had to cut down on her work in her private practice. She has episodic depression that lasts two to five months, characterized by low energy and lack of interest in doing things, decreased productivity, and increased absenteeism. Fortunately, her colleagues were understanding and were providing her with less stressful cases during these periods, allowing her to continue working. However, this mood volatility continued to impact her private practice, which caused her great uncertainty and instability in her income. She has recovered fairly well from the depressive episodes but continues to work apprehensively for the future because of a possible relapse into another depressive episode.

Case 8: Female, 27, Pharmacist

A 27-year-old female pharmacist was diagnosed with severe episodic long-term depression in 2021, which made her daily life and professional activity balance highly problematic. It was accompanied by anxiety for three to six months of duration and led to a sharp decline in productivity and an increase in absenteeism, which caused her to lose a full-time job. Although some colleagues have been supportive, the fundamentally poor attitude at work has oversalted this. She has subsequently taken part-time work but reports continuing feelings of inadequacy and failure at work and in the family. She now receives treatment but still feels in doubt as to whether she will be able to cope with her responsibilities and continue her working career.

Systematic review

Study Selection and Characteristics

Overall, 29 studies were included in this systematic review, with very considerable heterogeneity covering a wide diversity of countries, populations, and study designs (Table 3) [7-35]. The origin of these studies was from several settings, including the United States, Canada, Finland, Denmark, the United Kingdom, and many European nations, thus giving a general perspective on how chronic and episodic long-term depression influences employment outcomes. Longitudinal cohort study designs, cross-sectional analyses, and randomized controlled trials were identified within the reviewed articles to provide strong evidence for this. The samples ranged from small populations in the hundreds of participants to large nationwide cohorts with hundreds of thousands of subjects.

**Table 3:** Summary of key studies examining the impact of depression on employment and career outcomes. ACEs = adverse childhood experiences; BDI = Beck’s Depression Inventory; CCHS = Canadian Community Health Survey; CIDI = Composite International Diagnostic Interview; CIDI-SF = Composite International Diagnostic Interview-Short Form; CIDI-SFMD = Composite International Diagnostic Interview-Short Form for Major Depression; CES-D = Center for Epidemiologic Studies Depression Scale; DSM-IV = Diagnostic and Statistical Manual of Mental Disorders, Fourth Edition; ICD-10 = International Classification of Diseases, 10th Revision; MDI = Major Depression Inventory; MDD = major depressive disorder; MHI-5 = Mental Health Inventory-5; NEET = not in education, employment, or training; NPHS = National Population Health Survey; PHQ-2/PHQ-4/PHQ-9 = Patient Health Questionnaire-2/4/9; RTW = return to work; SCL-CD6 = Symptom Checklist Core Depression Subscale-6; SF-36 = 36-Item Short Form Health Survey; SMFQ = Short Mood and Feelings Questionnaire; WLE = working life expectancy

Study ID	Author	Country	Year	Study design	Sample size	Population demographics	Depression measurement	Career outcomes measured	Impact on career advancement	Impact on job stability	Follow-up duration	Key findings
1	Holma et al. [7]	Finland	2012	5-year prospective study	269	Adults with MDD, mean age 39.6, 72% female	DSM-IV MDD diagnosis	Disability pension rates	Older age (50+) and time spent depressed hindered career advancement	20% granted disability pension	5 years	Older age, time depressed, comorbidities, and lack of education predicted disability pension
2	Hakulinen et al. [8]	Denmark	2019	Nationwide cohort study	2,390,127	Individuals aged 15–25, 49% female	ICD-10 codes (F32-F33)	Employment, income, education	Early-onset depression reduces income and education across life	High unemployment rates in early adulthood	25–61 years	Severe mood disorders linked to poor socioeconomic outcomes
3	Chen et al. [9]	Taiwan	2023	Matched cohort study	420,935	Individuals aged 15–64, mixed gender	ICD-10 codes (F32-F33)	Employment status, income	Significant drop in employment and income post-diagnosis, more severe in older men	Employment rate dropped 8.1%, income by $2,006 after 5 years	10 years	Depression caused a substantial decline in employment and income, with lasting effects
4	Petersen et al. [10]	Germany	2022	Cross-sectional study	2,288	Adults aged 25+, mean age 53.3, 47.7% male	PHQ-2 (part of PHQ-4)	Education, employment, income	Depression worsened the economic impact of adverse childhood experiences	Higher unemployment, more blue-collar jobs in those with ACEs	N/A	Depression amplifies economic burdens linked to ACEs
5	Rizvi et al. [11]	Canada	2015	Multi-site retrospective/cross-sectional	986 (primary), 274 (tertiary)	Adults aged 18–75, mixed gender	DSM-IV MDD Diagnosis	Employment, absenteeism, disability rates	Anhedonia strongly predicted disability, affecting career	High unemployment and disability rates, absenteeism worsened by comorbidities	N/A	Severe depression and comorbidities lead to higher unemployment and disability
6	Ringdal et al. [12]	Netherlands	2022	Longitudinal study	7,283	Adults under 65, 45% male	MHI-5 Score	Labor force participation, full-time work	Severe depression reduced employment, especially in men	Higher unemployment and less full-time work in depressed men	10 years	Severe depression reduces employment, especially in men, with pessimism playing a role
7	Elinson et al. [13]	United States	2004	Cross-sectional study	2,166	Adults aged 18–69, mixed gender	Self-reported major depression	Labor force participation, work limitations	Depression reduced career advancement due to work limitations	High unemployment, significant work limitations impacting job stability	N/A	Severe depression linked to work impairment and reduced job stability
8	Gilmour et al. [14]	Canada	2007	Cross-sectional/longitudinal	17,433 (CCHS), 20,167 (NPHS)	Adults aged 25–64, mixed gender	CIDI (based on DSM-IV)	Work activity, disability days, absenteeism	Depression reduced work activity, increased disability days, especially in white-collar jobs	High absenteeism, reduced productivity in high-stress jobs	2–8 years	Depression causes work impairment, more severe in high-stress white-collar jobs
9	Andreeva et al. [15]	Sweden	2015	Longitudinal study	3,503	Swedish workers, mixed gender, aged 18+	SCL-CD6 Scale	Job loss, employment status, depressive symptoms	Job loss due to downsizing increased depression risk, with gender differences	Higher unemployment in women with pre-existing depression; surviving layoffs increased depression	2 years	Job loss during downsizing events increases depression risk, especially for women
10	Druss et al. [16]	United States	2001	Longitudinal study	6,239	Employees of 3 corporations, mixed gender	SF-36 Mental Component Summary	Sick days, work effectiveness, health care satisfaction	Depression lowered work effectiveness, increased absenteeism, especially with health care dissatisfaction	High absenteeism, reduced work effectiveness among chronically depressed employees	2 years	Chronic depression leads to more missed work days and lower work effectiveness
11	Witt et al. [17]	Australia	2021	Prospective cohort study	2,518	Adolescents aged 11–17 followed into adulthood	Short Mood and Feelings Questionnaire (SMFQ)	Education completion, employment, job stressors	High adolescent depressive symptoms reduced odds of education completion and employment	Lower employment, increased job stressors for those with high adolescent depression	~10 years	High adolescent depression decreases education completion and employment rates
12	Campbell et al. [18]	United Kingdom	2022	Mendelian randomization study	227,242	Working-age adults (40–64), white British ancestry	Genetic markers, self-reported depression	Employment status, hours worked, income, education	Depression increased non-employment due to sickness/disability with limited impact on other outcomes	Higher sickness/disability-related non-employment rates	5 years	Depression liability increases sickness/disability-related non-employment
13	Heinz et al. [19]	United States	2018	Longitudinal Study	424	Adults aged 40, mixed gender	Global Depression Scale	Employment status, occupational prestige, work environment	Higher job prestige and supportive work environments linked to less severe depression, better career outcomes	Lower work stress, better job stability with supportive environments	23 years	High job prestige and support at work reduce depression severity, aiding career stability
14	Thielen et al. [20]	Denmark	2013	Longitudinal study	5,785	Adults aged 40 and 50, mixed gender	Major Depression Inventory (MDI)	Job change, unemployment, sick leave, disability	High physical work demands and depression increased job changes, unemployment, disability	Increased sick leave, disability due to high physical demands and depression	7 years	High physical demands combined with depression increase job instability and disability
15	Prieto-Vila et al. [21]	Spain	2024	Longitudinal RCT	483	Adults aged 18–65, mixed gender	PHQ-9	Employment status, disability, symptom trajectories	Chronicity and relapse led to higher disability, lower employment, and poorer life quality	Higher unemployment, disability in chronic/relapsing depression	12 months	Chronic/relapsing depression linked to high disability and poor job outcomes
16	Patten et al. [22]	Canada	2009	Longitudinal cohort study	6,570	Adults aged 26–65, employed at baseline	CIDI-SF	Employment status, transition to nonworking status	Major depression doubled the risk of moving to non-working status, especially in ages 26–45	Increased unemployment and non-working status due to major depression, particularly in younger adults	10 years	Major depression increases the risk of unemployment, particularly in younger adults
17	Rottinghaus et al. [23]	United States	2009	Cross-sectional study	388	College students (18–24), 61.9% female	CES-D Scale	Career decision status, self-efficacy	Depression linked to lower career decision status and self-efficacy	Career decision uncertainty linked to higher depressive symptoms, instability in career paths	N/A	Career decision status is lower in depressed students, affecting career paths
18	Zivin et al. [24]	United States	2012	Longitudinal study	516	Veterans of working age (18–65), 94% male	PHQ-9	Employment status, changes in employment	Improved depression status increased job stability; persistent depression maintained unemployment	Persistent depression linked to ongoing unemployment; improvement increased job stability	18 months	Improved depression status increases job stability; persistent depression leads to unemployment
19	Naicker et al. [25]	Canada	2013	Longitudinal study	1,027	Adolescents aged 16–17, followed into early adulthood	CIDI-SFMD	Employment status, income, education, social support	Adolescent depression had limited direct impact on employment but linked to long-term health issues affecting career	Recurrence and severity of symptoms may contribute to future job instability	10 years	Adolescent depression linked to long-term health issues, indirectly affecting job stability
20	Lee et al. [26]	South Korea	2016	Longitudinal study	5,241	Adults aged 20+, mixed gender	CES-D 11	Work ability, employment status	Loss of work ability increased depression, leading to severe job instability	High depression scores linked to loss of work ability and employment	1 year	Loss of work ability increases depression, leading to job instability
21	Bubonya et al. [27]	Australia	2019	Longitudinal study	14,479	Working-age adults (18–64), mixed gender	MHI-5 Scale	Employment status, labor force participation, unemployment rates	Severe depression increased non-employment, unemployment, especially in men	Non-employment, unemployment linked to higher depressive symptoms, mainly in women	14 years	Severe depression significantly increases unemployment, with a stronger effect in men
22	Woodhead et al. [28]	United States	2020	Longitudinal study	382	Adults diagnosed with depression, 56% women	Self-reported depression severity	Employment status, work functioning, medical/social functioning	High-severity depression linked to poor work functioning, employment outcomes.	Decline in work and employment over 23 years in high-severity depression	23 years	High-severity depression worsens work and employment outcomes over time
23	Dobson et al. [29]	United States	2024	Longitudinal study	9,206	Adults aged 28–62, mixed gender	CES-D Short Form (CES-D-SF)	Working life expectancy (WLE), labor force status	Persistent high-symptom depression significantly reduced WLE and job stability	High-symptom trajectories linked to decreased job stability, shorter employment duration	30+ years	Persistent high-symptom depression shortens WLE and increases job instability
24	Lee et al. [30]	United States	2020	Longitudinal study	834	Adults aged 34–50, mixed gender	CES-D Scale	Employment status, income, depression trajectories	Depression followed a V-shaped curve; employment and net worth were protective	Employment stability and higher net worth linked to lower depression, especially at 40	16 years	Depression trajectories show non-linear change; employment and financial stability protect against severe depression
25	López-López et al. [31]	United Kingdom	2019	Longitudinal study	9,399	Individuals aged 11–24, mixed gender	Short Mood and Feelings Questionnaire (SMFQ)	University degree, NEET status at 24	Persistent high depression in adolescence reduced university degree attainment, increased NEET risk	High risk of NEET status and lower university attainment linked to persistent adolescent depression	13 years	Persistent adolescent depression decreases education and employment outcomes, increasing NEET risk
26	Lamberg et al. [32]	Finland	2010	Longitudinal cohort study	14,487	Adults aged 20–54, mixed gender	Beck's Depression Inventory (BDI)	Disability retirement, unemployment status	Severe depression increased disability retirement, especially in short-term unemployed	Higher disability retirement in unemployed, especially with severe depression	5 years	Severe depression increases disability retirement, particularly among unemployed
27	Lerner et al. [33]	United States	2004	Longitudinal study	489	Employed adults aged 18–62, mixed gender	PHQ-9, CIDI	Unemployment rates, job retention, presenteeism, absenteeism	Depression increased unemployment, job turnover, presenteeism, absenteeism	Higher unemployment and turnover in depressed employees compared to controls	6 months	Depression leads to higher unemployment, turnover, presenteeism, and absenteeism
28	Huijs et al. [34]	Netherlands	2017	Prospective cohort study	883	Long-term sick-listed employees, mixed gender	CES-D Scale	Return to work (RTW) status, work characteristics	Depression delayed RTW, especially with low decision authority and RTW self-efficacy	Lower RTW rates, longer sick leave in depressed employees	2 years	Depression delays RTW, especially with low decision authority and support
29	Veronese et al. [35]	Europe (29 countries)	2012	Cross-sectional study	31,126	Working-age adults (18–70) across 29 European countries	Composite International Diagnostic Interview (CIDI)	Work disability, employment status, health status	Depression increased work disability, especially in older adults, women, and those with comorbid conditions	High work disability and non-employment, especially with comorbidities	1 year	Depression significantly increases work disability, particularly among vulnerable groups

Impact on Career Advancement

The findings consistently showed that chronic and episodic long-term depression both have negative implications on career development. For example, Holma et al. (2012) [7] reported that older age and longer duration of depression were strong predictors for being awarded a disability pension, which stops any kind of promotion. Similarly, Hakulinen et al. (2019) [8] conducted a nationwide cohort study in Denmark with a population of more than 2.3 million individuals. The study identified that early-onset severe mood disorder was the most important predictor of lower income and educational attainment, underlying persistent socioeconomic disadvantages across life. This very early career development disruption is a long-term consequence of depressive disorders on socioeconomic status. Chen et al. (2023) [9] reported that employment rates and incomes were significantly reduced following a diagnosis of depression in Taiwan. The employment rates decreased by 8.1% within the five years following initial diagnosis, with a mean decrease in income of USD 2,006. These effects were more striking among older age groups and men, showing a compounded disadvantage in these demographics. Petersen et al. (2022) [10] showed how depression makes adverse childhood experiences a little worse for job promotion. The study established that there is a strong relationship between the increased events of adverse childhood experiences and more severe symptoms of depression. This, in turn, influences reduced educational achievements and decreased income. In this case, not just the direct effects on career outcomes but also acting as amplifiers of early-life adversities might be associated with depression.

Impact on Job Stability

Depressed patients were primarily concerned about job stability. Across all studies, depression was significantly linked to high levels of unemployment, job turnover, and work incapacity. Rizvi et al. (2015) [11] investigated the impact of core symptoms, especially anhedonia, on high rates of unemployment and disability in Canada. Comorbid conditions more significantly worsened such outcomes, entailing tremendous job instability. In the Netherlands, Ringdal et al. (2022) [12] emphasized that men with severe depression were less likely to maintain full-time employment. The study suggested that pessimistic beliefs and lower life satisfaction, often associated with severe depressive symptoms, played a mediating role in reducing labor force participation and increasing job turnover. Lerner et al. (2004) [33] presented data from the United States showing that subjects with depression had higher levels of unemployment, job turnover, presenteeism, and absenteeism than non-depressed controls and those with rheumatoid arthritis. These results point to a ubiquitous effect of depression on job retention and the ability to hold a steady job. On the other hand, Huijs et al. [34] assert that in 2017, which was supported by the finding that depressive symptoms were predictive of a longer time to fully return to work. It has also been shown that workers with greater physical demands in their jobs are vulnerable to long-term sick leaves and less likely to return to their old jobs.

Socioeconomic Consequences

The socioeconomic implications of depression were severe and multidimensional in terms of individual economic stability and broader societal productivity. Druss et al. (2001) [16] showed that chronic depression was strongly predictive of poor work outcomes at follow-up two years later, specifically within a subgroup of those dissatisfied with their healthcare. This would probably have increased the impact of depression further by reducing productivity and job performance. A recent study by Witt et al. (2021) [17] discovered how core symptoms of depression during adolescence considerably reduced, or altogether eliminated, the possibility of finishing secondary education, consequently affecting employment opportunities during young adulthood. It was also reported in this Australian study that the first group was more exposed to several different factors of job stressors, including workplace incivility, which had probably further increased the risk of depressed individuals having their depressive symptoms exacerbated and hindered career advancement.

In a large cross-sectional study across 29 European countries, Veronese et al. (2012) [35] identified that depression significantly increased the likelihood of work disability. This effect was particularly pronounced among older adults, women, and those with comorbid conditions such as angina. The study highlighted the complex interplay of demographic and clinical factors that contribute to work disability in individuals with depression, indicating that certain populations are more vulnerable to the economic impacts of the disorder.

Long-Term Effects

Heinz et al. (2018) [19] gave it a temporal dimension by showing that higher occupational prestige and more supportive work environments are tied to less intensive or severe trajectories of depression, which, in turn, improves career advancement over 23 years. It appears that during a depressive state, negative effects on career progression could be offset by the presence of a supportive work environment and higher job prestige. In addition, Prieto-Vila et al. (2024) [21] identified that chronicity and relapse in depressive symptoms were associated with higher disability rates and a lower likelihood of employment. The study, conducted over a 12-month period in Spain, found that individuals with chronic or recurrent depression were more likely to experience job instability, highlighting the importance of early intervention and continuous support for those at risk of long-term depressive episodes.

Lee et al. (2020) [30] followed a trajectory curve of depressive symptoms over time, reporting a V-shaped curve that showed higher levels at ages 34 and 50. Obtained evidence suggested employment stability and financial security as significant protection factors against severe depression, emphasizing that stable employment is important in mitigating the long-term effects of depression.

Gender Differences

Gender differences in the impact of depression on employment outcomes were evident across the studies. Andreeva et al. (2015) [15] found that job loss due to downsizing significantly increased the risk of major depression, with women who had pre-existing depression being more likely to be laid off. This finding suggests that women may face additional challenges in maintaining employment when experiencing depressive symptoms, particularly during periods of economic downturn. Bubonya et al. (2019) [27] suggested that it is on the contrary, with more severe depressive symptoms having the greatest impact on men. The Australian study reported that though the male category was more affected by non-employment due to depression, the association of labor market status with depressive symptoms was weaker for women.

Summary of Findings

Overall, these studies have highlighted that chronic and episodic long-term depression affect both career advancement and job stability. While some of these adverse outcomes may be tempered by more supportive work environments and greater job prestige, those with more severe or chronic depressive symptoms still bear an increased risk of job instability and economic disadvantage. Moreover, gender differences only further complicate things, calling for focused interventions to address the different problems that men and women experiencing depression encounter in the workplace.

## Discussion

Major depressive disorder is considered to be one of the leading contributors or causes of global health burden, affecting an estimated 300 million sufferers from the disorder worldwide and accounting for 40.5% of years lived with disability caused by mental and substance use disorders [36]. There has been considerable change in the concept of depression since the latter part of the 18th century. Initially, *melancholia* was a disorder of intellect, characterized by delusions and partial insanity. At the end of the 19th century, with Griesinger, Maudsley, and Kraepelin, the emphasis shifted to redefine melancholia primarily as a mood disorder. This change in emphasis furnished the background for our current notion of depression as an emotional and mood disorder and not an intellectual malfunction [37]. This study provides a comprehensive analysis of the impact of chronic and episodic long-term depression on employment outcomes, drawing insights from both a systematic review and a series of detailed case studies. The findings underscore the significant challenges that individuals with these forms of depression face in maintaining stable employment, advancing their careers, and achieving financial security.

Besides these correlations of depression with career difficulties, several mechanisms contribute to these challenges. Workplace stigma is a major mechanism wherein persons suffering from depression are discriminated against or perceived as incapable, thereby affecting their career advancement or leading to job loss. Matters are worsened as most workplaces lack the much-needed mental health support, where workers with depression get neither accommodation nor interventions that may make them productive. It is further worsening with systemic barriers erected against those attempting to re-enter the workforce after a period of unemployment due to depression, such as lack of access to re-employment programs and social safety nets. Mitigating these factors is an essential part of creating a supportive work environment and overcoming the career-related impact of depression [34].

There was a clear association between depression, lower income, higher unemployment rate, and increased disability claims in different populations and settings. For instance, Holma et al. [7] in 2012 and Hakulinen et al. [8] in 2019 indicated that older age and early-onset depression are associated with poor career progression, which generally results in leaving the workforce at an early age. These findings are supported by the case series, whereby most of those reporting severe depressive episodes lost their jobs, had reduced productivity, and financial difficulties.

The narratives of the eight cases highlighted not only the direct impact of depression on job performance but also the role of workplace support in either mitigating or exacerbating these effects. For example, Case 2 demonstrated how a lack of understanding from colleagues can worsen the impact of depression, leading to significant career disruptions. Conversely, Case 7 showed that a supportive work environment could help maintain job stability, even in the presence of severe depressive episodes. Thus, the review concluded that depressive symptoms, more so severe or recurrent ones, are career-relaunching. This would be more pronounced in studies such as that by Chen et al. (2023) [9] on the post-diagnosis decline of employment rate and income seen more in older men. This was mirrored in the case series, with many reporting stalled career growth, an inability to hold multiple jobs, and withdrawal from the workforce in the most tragic cases. Job stability is a serious issue, as depression has been associated with increased job turnover, absenteeism, and long-term unemployment. The review provided robust evidence of this, with studies such as those by Ringdal et al. (2022) [12] and Lerner et al. (2004) [33] demonstrating that individuals with depression are more likely to experience frequent job changes and longer durations of unemployment. The case series further contextualized these findings, showing that these outcomes are often compounded by factors such as lack of social support, comorbid conditions, and workplace stigma.

The socioeconomic effects of depression are deep and multi-faceted. Indeed, the systematic review strongly pointed out that those with depression are more likely to be plunged into poverty because of the inability to sustain stable employment together with the related financial demands. This is supported by the case series where almost all cases reported significant reductions in income with depletion of savings as a consequence of their condition. Gender differences were also evident in the impact of depression on employment outcomes. The systematic review found that while both men and women are adversely affected, the nature and extent of these effects differ. The case series supported these findings, with women more frequently reporting a complete withdrawal from the workforce and men reporting slower career progression due to depressive symptoms.

Treatment for depression has evolved considerably over the years, but traditional pharmacological approaches remain central, mainly represented by antidepressants targeting serotonin, norepinephrine, and dopamine. Even with these treatment options, many patients do not reach full remission, especially those diagnosed with treatment-resistant depression. Recent developments have enriched this armamentarium with new tools, including ketamine and its derivative esketamine, acting on NMDA receptors and providing fast relief sometimes within hours. Transcranial magnetic stimulation has also gained recognition for its efficacy in treatment-resistant depression by non-invasively stimulating specific brain regions. Additionally, psychedelic-assisted therapy, particularly with psilocybin, has shown promise in treating depression by increasing brain plasticity and serotonin receptor activity. Lastly, anti-inflammatory agents are being explored as adjunctive treatments, particularly in patients whose depression is linked to inflammatory processes. These emerging treatments represent significant advancements, offering hope to those who have not responded to traditional therapies [38].

Results from this study further highlight how chronic or episodic long-term depression significantly affects employment outcomes. Systematic review results are combined with qualitative case studies to elucidate the complex interplay of how depression interacts with personal, workplace, and societal influences on employment. It is these insights that underline the acute need for such focused interventions to deal with the psychological and social factors of depression, which would help an individual sustain his or her employment and improve their quality of life.

This study has several limitations that should be noted. The case series, while providing detailed insights, involves a small sample size of eight individuals, which may limit the generalizability of the findings. Additionally, the reliance on self-reported data in the case series may introduce bias. The systematic review, although comprehensive, is subject to the variability and quality of the included studies. Differences in study designs and measures of depression and employment outcomes may affect the consistency of the results. This study provides important insights into the relationship between depression and employment outcomes, despite its limitations. Future research should investigate workplace interventions that target enhancing support, reducing stigma, and providing individually tailored help to people affected by depression, particularly those in a precarious position concerning job instability and socioeconomic decline.

## Conclusions

This study highlights the significant impact of chronic and episodic long-term depression on employment outcomes, including career advancement, job stability, and socioeconomic well-being. The findings from the systematic review and case studies reveal that depression frequently leads to reduced productivity, absenteeism, and job loss, often compounded by a lack of workplace support and societal stigma.

These challenges can have long-term consequences not only for the worker but also for the economy he/she is a part of and broad societal factors. Future studies should focus on developing interventions targeted toward support enhancement at work, reducing stigma, and offering appropriate support to those at the very highest risk levels of job instability and financial decline. Many of these needs can be realized to enhance the quality of life for people with depression and reduce its burden on society as a whole.
